# Visual Expression of Emotion in Dynamic 3D Painting System Based on Emotion Synthesis Model

**DOI:** 10.3389/fpsyg.2021.730066

**Published:** 2021-08-19

**Authors:** Shenghe Cheng

**Affiliations:** College of Art, Nanjing University, Nanjing, China

**Keywords:** emotion synthesis model, dynamic three-dimensional painting system, emotion visualization, discrete method, emotional synthesis model

## Abstract

Emotion is a unique ability possessed by human beings as advanced creatures. Emotions give people a unique physical and mental experience. Assigning emotions to computer systems is one of the latest topics in artificial intelligence research. The purpose is to allow machines to achieve natural coordination between humans and computers. This article focuses on the visual expression of emotion in the dynamic three-dimensional painting system, creating an intelligent painting system and realizing a good user experience. In this paper, the discrete method is used to qualitatively analyze emotions, and the continuous method is used to quantify basic emotions, and emotional modeling and emotional quantitative analysis are proposed to realize quantitative analysis of emotions. Combining these two methods, a comprehensive method is proposed, which uses a continuous method to quantify the basic emotions of each discrete dimension, and finally superimposes them into a comprehensive emotional synthesis model. Emotion modeling is the basis of emotion visualization. Borrowing the relationship between emotion synthesis model and visual emotion elements, this article puts forward the concept of qualitative and quantitative visual emotion elements, and expounds that the multidimensional superposition of visual emotion elements makes dynamic three-dimensional painting system emotions. The experimental results in this article show that the emotional visualization scheme of 100 samples is tested by quantitative statistical methods to demonstrate its effectiveness. Starting from 5 points of concern, the emotion visualization method discussed in this article can indeed convey or suggest a certain positive emotion (the average value of experience, transitivity, and infectiousness > 2.5, and the variance is close to 0), but we also found this recognition at the same time The degree is not high enough, and individual differences are large (mean value < 2.5, variance close to 1). This can indicate that different subjects have different feelings and evaluations of this emotional visualization. As long as the difference is within a reasonable range, this emotional visualization also has practical value, and has the ability to convey or suggest emotions.

## Introduction

### Background

In recent years, computer networks have developed rapidly and have relatively mature functions, which have produced many amazing applications. However, current computers still have some shortcomings. For example, computers cannot make optimal decisions like humans. Although computers have huge computing power and storage capabilities, they lack the ability to perceive or express emotions, that is, a certain human emotional mechanism (Tang and Elhoseny, 2019). Therefore, in order to better realize the human-computer interaction, strengthen the emotional communication between users and the system in the dynamic 3D painting system, use the dynamic 3D painting system as the research object, and try to model abstract emotions through emotional calculations, effectively expressed in a dynamic three-dimensional painting system.

### Significance

With the advancement of science and technology and the development of living materials, people’s requirements for smart computers are getting higher and higher. If a computer has functions similar to human emotion mechanisms, then it will be smarter and more suitable for interacting with users. For example, a computer that can sense the user’s drowsiness should not endlessly instill more information to the user. If computers can eliminate the estrangement between people and have a certain emotional mechanism like normal people, human-computer interaction will be another scene, just like a universal housekeeper. In practical applications, this is exactly what the research fields including artificial intelligence and pervasive computing want to achieve, and it is also what many website designs are trying to achieve today (Wang and Lu, 2019). By modeling emotions, computers can learn to analyze and express human emotions, have a certain emotional mechanism, eliminate obstacles between computers and humans, and provide better services. Therefore, a three-dimensional website with an emotional mechanism will greatly enhance the user experience of the website and enhance the superior experience of human-computer interaction.

### Related Work

Emotion is considered to be the basic element of human-computer interaction performance. In expressive synthesized speech, it is important to generate emotional speech that reflects subtle and complex emotional states (Liu and Fu, 2021). However, the research on how to effectively synthesize emotional speech through intuitive control using different levels of emotional intensity is limited, and it is difficult to model effectively. Zhu and Xue (2020) explored an expressive speech synthesis model that can be used to generate speech with a variety of emotional strengths. Different from the previous study of encoding emotions into discrete codes, he proposed an embedded vector to continuously control the intensity of emotions. This is a data-driven method that can synthesize speech through fine control of emotions. Compared with models using retraining techniques or one-hot vectors, his model using embedding vectors can clearly learn high-level emotional intensity from low-level acoustic features. Therefore, he can control the emotional intensity of synthesized speech (Zhu and Xue, 2020). When people face internal or external stimuli, emotions are a subjective and conscious experience. Qiu and Zhao (2018) research solves the problem of emotional computing that is difficult to intuitively apply to practical areas in the real world due to unintuitive data representation, such as the diagnosis of emotional diseases. Given that people’s ability to understand two-dimensional images is much higher than that of one-dimensional data, he uses Markov transition fields to visualize time series signals. The MTF image represents the first-order Markov transition probability along one dimension and the time dependence along another dimension. In addition, due to the limitations of experimental equipment and individual differences between volunteers, noise is inevitable. He applied AC-GAN to remove noisy pixels in high dimensions and obtain high-resolution images before classification. Then he used Tiled Convolutional Neural Networks on 2 real-world data sets to learn advanced features from MTF images. The classification results of other methods are competitive with the most advanced methods. This method makes it possible to recognize emotions based on visualization, which is conducive to the application of cognitive robots or the diagnosis of psychological problems such as depression in the medical field, and can help doctors and patients understand the condition more intuitively (Qiu and Zhao, 2018). Yu et al. (2017) believes that the emotional appeals that prevailed in charts and graphs in the late nineteenth century have basically been dormant since then and quickly reappeared in contemporary data visualization. This new form of data design changes the relationship between designers and users, and strengthens the emotional impact of the data by triggering emotions ranging from excitement and empathy to anxiety and fear. Yu et al. (2017) provided a historical overview of the tragic appeal in data design in the late nineteenth century and the transition to modernist minimalism in the twentieth century. Contemporary examples from companies, non-profit organizations, government agencies, and individual designers illustrate how data visualization can stimulate emotions, and the results of the research show that the emotional appeal of the nineteenth century is concentrated (Yu et al., 2017). Although the research perspective is forward-looking, there are still some shortcomings in research techniques and methods.

### Innovation

The innovation of this article lies in (1) By acquiring emotional data, an emotional synthesis model is established to effectively analyze and express emotions. Calculate emotions qualitatively and quantitatively, and embody abstract emotions. It is measured by certain variables (such as prompts and incentives) and induces variables. (2) Based on the relationship between the emotion synthesis model and visual emotion elements, explore ways to visualize emotions. The concept of qualitative and quantitative visual emotion elements is put forward, and the multi-dimensional superposition of visual emotion elements enables the three-dimensional dynamic website space to express complex emotions.

## Research Foundation

### The Mechanism of Emotion Generation

**FIGURE 1:**
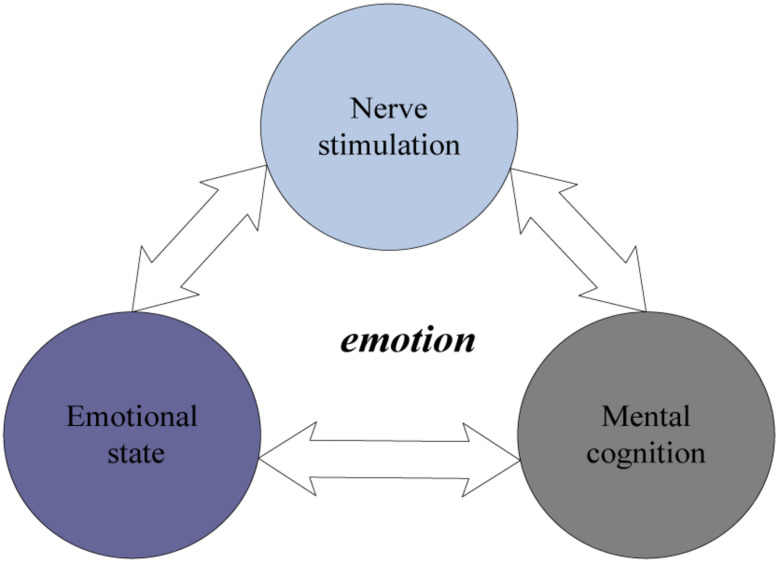
The mechanism of emotion generation.

The key to the mechanism of emotion generation is the physiological and psychological mechanisms, including the neural stimulation layer, the emotional state layer and the psychological cognitive layer. The three are closely related, that is, under the influence of nerve stimulation and hormones, the corresponding emotional response is generated, and the body works together Produce emotion (Ridderinkhof, 2017). Through the study of the basic mechanism of human emotions, the understanding of human emotion mechanisms, and the use of direct emotional stimulation and information injection of mental cognitive abilities, will help us to further study how to concretize and model complex and abstract emotions. Can design a dynamic three-dimensional painting system that can stimulate people’s emotions. Emotional audio-visual elements enable us to inject emotional information into user consciousness to achieve emotional visualization (Huxtable-Thomas et al., 2016). Visual and auditory stimulation is the most effective way to stimulate the cognitive level. For example, seeing a black room feels depressed, and hearing a joke feels joy. We can use emotional audio-visual elements to try to create a dynamic three-dimensional painting system that visualizes emotions. The mechanism of emotion generation is shown in Figure 1.

### Emotion Synthesis Model

#### Modeling Process

**FIGURE 2:**
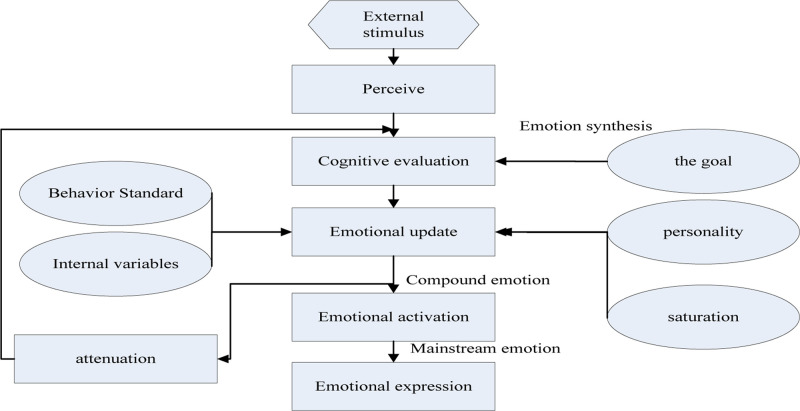
Modeling process of emotion synthesis model.

Psychologists define the various possible factors that can cause changes in human emotions as evaluation factors (including the degree of expectation, relevance, adaptability, causes, etc.), and put forward the theory of emotional evaluation. It is believed that individuals can only produce emotions by cognitive evaluation of stimuli. There are also personality psychological structures, such as interest, motivation, and personality characteristics, which also have an impact on the generation of emotions (Topal and Ozsoyoglu, 2017). Individuals of different personalities have different evaluations of the same stimulus. The resulting emotions are also different. In addition, psychological experiments prove that when the emotional intensity reaches a certain level. Even if there are constant external stimuli, the intensity will not continue to increase, that is, emotions will reach saturation, especially after some basic emotions are activated, even if there is no external stimulus, they will not disappear immediately. It will gradually decay over time (Ren and Matsumoto, 2017). This article is based on the motivation theory and cognitive evaluation of human emotion. It not only considers the impact of cognitive factors on emotions, but also non-cognitive factors (such as physiological needs, personality factors, the characteristics of emotions themselves, the mutual influence between emotions, etc.). Impact on emotions. The construction process is shown in Figure 2.

#### Basic Principles

**FIGURE 3:**
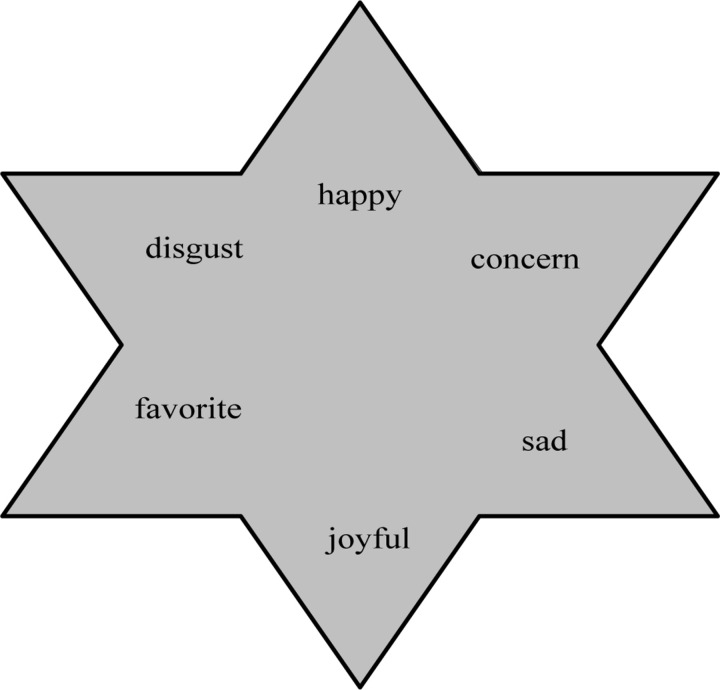
Discrete emotion synthesis model.

Discrete method of emotional synthesis. There are many ways to divide emotions in psychology (Eyben et al., 2017). Researchers have different definitions of emotion category labels, and Table 1 summarizes them.However, using discrete methods to construct complex emotions also has its limitations. On the premise of our six basic emotions, happiness, sorrow, sorrow, happiness, love, and evil, as shown in Figure 3, human emotions are divided into three dimensions: happiness, sorrow, joy, and love and evil, so as to achieve Qualitative analysis of emotions.

**TABLE 1:** Different researchers’ definitions of emotion category labels.

Researcher	Emotion category label
Ekman	Angry, disgusted, frustrated, happy, sad, surprised
Fridja	Desire, happiness, concern, surprise, curiosity, sorrow
Plutchik	Anger, identification, expectation, happiness, disgust, fear, sadness, surprise
Oatley	Anger, disgust, anxiety, happiness, sadness
Watson	Fear, love, anger
Graham	Happy, sad

Assuming that it represents the emotional dimension of happiness and worry, when j = 1, 0, −1 represents the dimension of happiness, without the dimension of anger and the dimension of anger, s represents the dimension of sadness and happiness, and h represents the dimension of love and evil. The combination of

(1) B=j∪s∪h,(j,s,h∈{-1,0,1})

In the above formula, B represents emotion; j, s, h represent various emotional dimensions. However, it is impossible for human emotions to be interpreted using such a simple model. We know that human emotions are extremely rich and cannot be fully described by a simple arrangement of 27 emotions.

**FIGURE 4:**
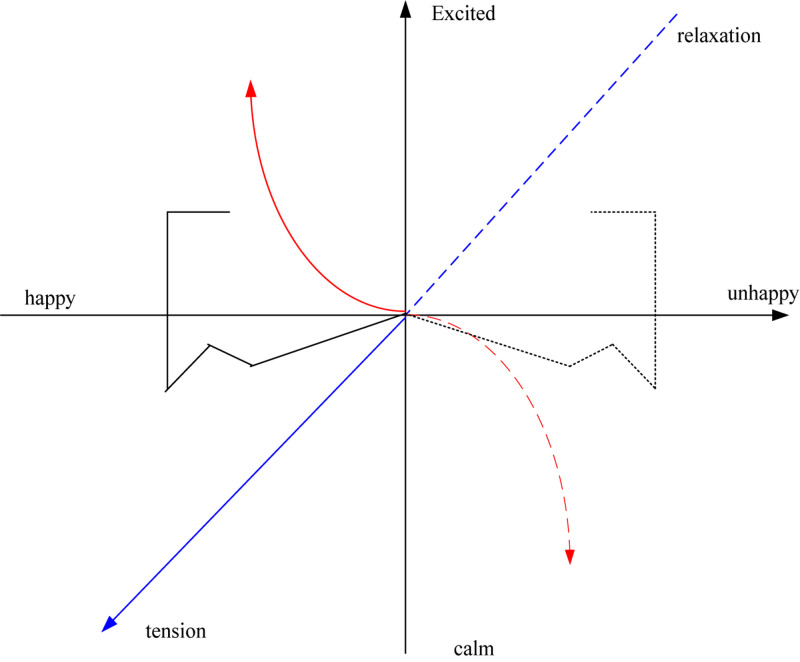
Wundt’s three-dimensional emotional model.

The dimensional method of emotion synthesis, also known as the continuous method of emotion synthesis, realizes the quantitative analysis of emotions (Radojevic, 2016). Due to the complexity of emotion generation and expression, many scholars have studied how to model emotions. Two three-dimensional emotional models are introduced here. The first is that the psychologist Wundt first proposed the theory of Emotion dimension. He researched and proposed that human emotions are composed of three dimensions, and different emotions will have two opposite directions in each dimension. Constantly making changes, namely excitement to inhibition (excitement-inhibition), pleasant to unpleasant (pleasure-displeasure), tension-relaxation (tension-relaxation) (Zhou et al., 2020). As shown in Figure 4.

**FIGURE 5:**
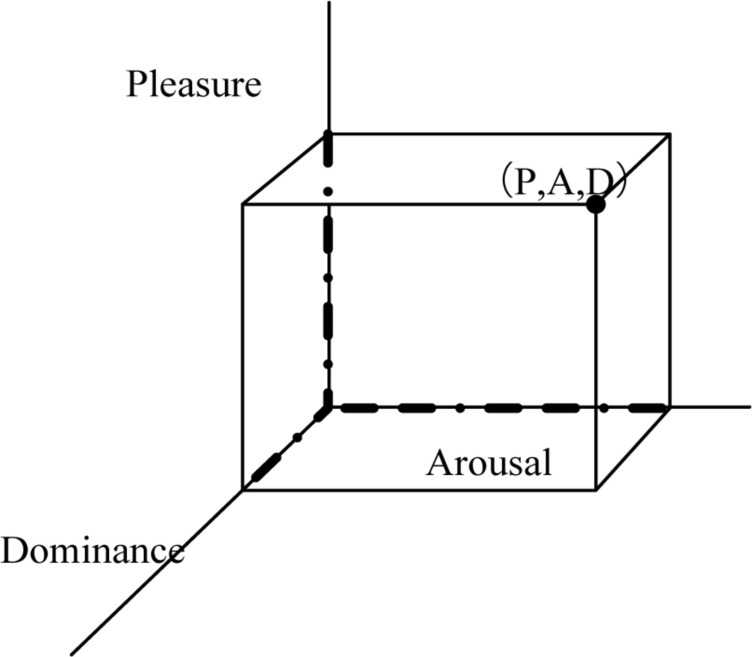
PAD three-dimensional emotional model.

The second type, in 1974, Mihrabian and Russell proposed a dimensional observation model-PAD three-dimensional emotional model. Take Figure 5 as an example. The three-dimensional emotion model of PAD is a kind of pleasure Pleasure, activation degree Arousal, dominance degree, and it divides emotion into three dimensions. Pleasure degree P describes the positive and negative characteristics of an individual’s emotional state, activation degree A describes the physiological activity of the individual’s nerves, and specifically quantifies the calmness and excitement of emotions, while dominance D describes the individual’s control over others and the situation, reflecting emotional control and control. The degree of Kim et al. (2016) and Hu et al. (2019).

Both discrete emotion model and dimensional emotion model have advantages and disadvantages. The two emotion synthesis models are compared (Ueoka and AlMutawa, 2018). As shown in Table 2:Comprehensive emotional synthesis. Combining the above two methods, a comprehensive method is proposed, which uses a continuous method to quantify the basic emotions of each discrete dimension, and finally superimposes it into an emotion synthesis model (Kostelnick, 2016; Volante et al., 2016). The formula of the comprehensive emotional model is as follows:

(2) $$E=f_{a-b}\,\left(u_{1},v_{1}\right)\cup f_{c-d}\,\left(u_{2},v_{2}\right)\cup f_{e-g}\,\left(u_{3},v_{3}\right),-1\leq u\leq 1,0\leq v\leq 1$$

**TABLE 2:** Comparison of two emotion synthesis models.

	Discrete emotion model	Continuous emotion model
Model complexity	Concise and easy to understand	More complicated
The way of emotional description	Independent adjective label	Coordinate point in space
The ability of emotional description	Single description with limitations	Arbitrarily complex emotion types
Advantage	Simple model, easy to start	Strong emotional description ability
Disadvantage	Limited emotional labels are difficult to satisfy	The process of quantifying complex emotions into specific values
	Description of mixed continuous emotions	Is arduous and the quality is difficult to guarantee

The function formula under the joy and worry dimension:

(3) $$f_{a-b}\,\left(u_{1},v_{1}\right)$$

The function formula under the sorrow dimension:

(4) $$f_{c-d}\,\left(u_{2},v_{2}\right)$$

Function formula under the dimension of love and evil:

(5) $$f_{e-g}\,\left(u_{3},v_{3}\right)$$

In the above formula, E represents emotion, U represents the incentive value of emotion, and V represents the induced value of emotion. The process of the value of u transitioning from −1 to 1 represents the process of transitioning from a negative emotion to a positive emotion, and the process of transitioning the value of v from 0 to 1 is also the process of the emotional activation degree rising to the full activation state (Huang, 2018; Wang et al., 2019).

The realization of the emotion synthesis model requires us to determine the basic attributes of each discrete dimension of emotion and how to combine the attributes of these discrete emotions. One of the most important steps to concretely realize this emotion model is that we need to set it based on the current visual emotion element under study. Here we set W1 as the value of the corresponding visual emotion element attribute on the basic emotion i1. At the same time, we define Si1 as a constant related to the basic emotion i1, which defaults to the value set during the emotional qualitative process (Kostelnick, 2016). We need to define some basic attributes of the emotional dimension, and the identification of these basic attributes is derived from the actual human emotions. Human emotions are composed of many basic emotions. However, the strength of each basic emotion is often different. That is, we need to assign a weight attribute to each basic emotion. Here we set the variable Zi1 as The weight attribute of the basic emotion. After defining many variables of the basic emotion of appeal, we need a way to mix emotions of various dimensions to obtain a model of complex emotions (Fortin and Cooperstock, 2017). For the basic emotions i1 and i2, then there are the following formulas:

(6) $$i=f\,\left(s,u,v\right)=\left[{\begin{array}{c} S_{i\,1}\,S_{i\,2}\\ a_{i\,1}\,a_{i\,2}\\ v_{i\,1}\,v \end{array}}_{i\,2}\right]∙\left[\begin{array}{c} \frac{Z_{i\,1}}{Z_{i\,1}+Z_{i\,2}}\\ \frac{Z_{i\,2}}{Z_{i\,1}+Z_{i\,2}} \end{array}\right]$$

In the formula, i represents the composite emotion of two emotional dimensions; S represents the constant related to emotion i; Z represents the weight of emotion i. This kind of emotion synthesis method can balance the emotions of different dimensions according to their weights, thereby synthesizing the corresponding complex emotions. According to the synthesized value, we can determine the synthesized value of the corresponding emotion, and then apply it to the visual emotion elements. Above, we can set the attributes of a single visual emotion element deterministically (Bringmann et al., 2016). For synthetic emotion, its weight satisfies the following formula:

(7) $$Z_{i}=Z_{i\,1}+Z_{i\,2}$$

Z represents the weight of emotion i, where the formula for calculating emotion intensity is as follows:

joy:

(8) $$M_{J\,O\,Y}=\left|1.7\times D\times E^{0.5}-\left(0.7\times D\right)\right|$$

sad:

(9) $$M_{d\,i\,s\,t\,r\,e\,s\,s}=\left|2\times D\times E^{2}-D\right|$$

hope:

(10) $$M_{h\,o\,p\,e}=\left|1.7\times D\times E^{0.5}-\left(0.7\times D\right)\right|$$

worry:

(11) $$M_{f\,e\,a\,r}=\left|2\times D\times E^{2}-D\right|$$

relief:

(12) $$M_{r\,e\,l\,i\,e\,f}=\left|f\,e\,a\,r\times D\right|$$

Disappointed:

(13) $$M_{d\,i\,s\,a\,p\,p\,o\,i\,n\,t\,m\,e\,n\,t}=\left|h\,o\,p\,e\times D\right|$$

In the formula, M represents the intensity of emotion, D is desirability, the satisfaction value, E is expectation, the expected value, and the expected value of the event is affected by two factors, namely, the impact of the event on the target and the importance of the target. The impact of the event on the target uses a fuzzy set {Extremely negative impact, relatively negative impact, no impact, relatively positive impact, extremely positive impact}, the domain value is [−1, 1], where 1 represents extremely negative impact, and 1 represents extremely positive impact. Goal The importance of is also described by a fuzzy set {not important, slightly important, obviously important, extremely important, and the domain value is [0, 1], where 0 represents unimportant, and 1 represents the expected value of extremely important events. The same applies to fuzzy sets {Extremely Unexpected, Less Expected, Neutral, More Expected, Extremely Expected} to describe, the domain value is [−1, 1], where −1 represents extremely undesirable, and 1 represents extremely expected (Liu et al., 2016).

**FIGURE 6:**
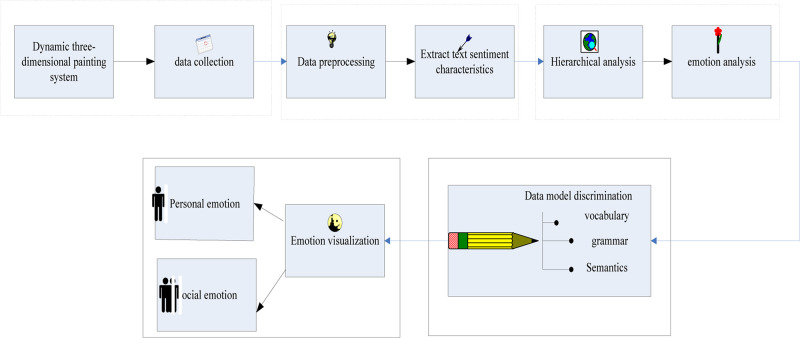
Emotion visualization process model.

The emotional visualization process model is shown in Figure 6:

The first step is to collect information, and the second step is to preprocess the information to extract the multi-dimensional attributes and emotional characteristics of the dynamic 3D painting system data. The third step uses the computational model to conduct a scientific system analysis of the dynamic 3D painting system. The fourth step is to perform visualization operations based on a specific data model, and the fifth step is to visualize emotions, including showing the changing trends of group users’ attitudes toward dynamic 3D painting systems under different characteristics for individuals and society.

### The Mechanism of Visual Influence on Emotion

Emotional changes are produced in the process of interaction between visual elements and the subject. Therefore, the key to emotional visualization is that the visual expression elements and expression forms of emotion are derived from the influence of visual information on emotions. After a long period of human evolution, the visual system has evolved a variety of neural mechanisms to adapt to the surrounding environment, each of which can efficiently process specific visual information. These specific visual information include position, form, texture, and movement (Kim et al., 2016; Zhang X. et al., 2020). In addition, the processing of visual information has the characteristic of filtering, which is accompanied by the immediate focus of human attention. The processing of visual information is carried out as a whole, and the combination of all the characteristics of visual perception and the characteristics of visual filtering constructs a visual perception that conforms to human psychology and habits. Psychology believes that the physiological principle of emotion is a person’s stressful body reaction directly caused by external stimuli, and this body reaction is directly produced by the nervous system. Not only that, vision is an important way for humans to obtain cognitive information and an important basis for human cognitive ability, which is also an important source of emotion. Therefore, the influence of visual information on emotion is obvious.

The current computer does not have the human emotion mechanism and the ability to think, and the construction of our three-dimensional website on the computer is also limited by this. Therefore, it is necessary to determine the basic emotional characteristics of these visual elements on the basis of the emotional synthesis model. What needs to be explained is that only when multiple visual emotional elements are combined to express emotions can we get closer to expressing emotions. Such as material elements, sports elements, color elements, styling elements, language elements, etc. Before realizing the construction of visual emotional elements, we need to define the visual characteristics of emotions, that is, the qualitative visual characteristics of emotional elements (Andrew and Larceneux, 2019; Zhang Y. et al., 2020). For example, blue represents sad emotions, red represents love emotions, and cyan represents evil emotions. Secondly, it is necessary to determine the specific value of the emotional characteristics of visual elements under different inducing forces and incentive values, that is, the emotional quantification of visual elements. For example, setting blue is the qualitative color of the emotional element of sadness. However, it is impossible for all sadness to be pure blue. We must specifically combine the emotional synthesis model, so that we can more accurately determine the specificity of this blue. The color value may eventually be found to be biased toward cyan. After that, the visual emotion elements of all dimensions are multi-dimensionally superimposed and calculated to finally generate the final dynamic 3D painting system, which is the final synthesis after qualitative and quantitative emotions, and we obtain a 3D emotional space of complex emotions (Topal and Ozsoyoglu, 2017).

## Experimental Process

### Purpose of the Experiment

The purpose of studying the emotional visualization expression method of the dynamic three-dimensional painting system is to convey emotions to the users of the system, and ultimately improve the user experience of the system, and propose corresponding solutions. Here, we specifically propose a visual expression method for emotions in this dynamic three-dimensional painting system. Verify that the method achieves our expected goals: First, does this emotional suggestion improve the user experience of the system? Second, whether the corresponding visual emotion element implies an emotional tendency.

### Subjects

This experiment is the first optimization screening of the visual expression method of emotion in the dynamic three-dimensional painting system. Using the method of random sampling, 100 college students were recruited to participate in this experiment (51 boys and 49 girls), aged between 18 and 25 years old, in good health, no defects such as color blindness, no mental illness, and similar Has a knowledge background, has a full understanding of new things, and is willing to accept new ideas and things. All subjects used individual tests and were divided into four groups according to different grades. After the experiment, the data was checked on the spot to ensure the validity of the experimental data.

### Experimental Method

The specific detection method is to first select multiple samples of the tested object, and experience some related states after experiencing this emotionalized 3D dynamic website. To achieve the purpose of emotion detection, we preset a number of detection concerns, including: the emotional state of the object, the transmission of website emotions, the experience of the website, the contagiousness of website emotions, and the acceptance of three-dimensional websites. After the subject completes this emotional visualization experience, they will use questionnaire surveys to collect answers to related questions regarding these concerns. As shown in Table 3, for each point of interest, five values are used to scale the value of the corresponding point of interest. A smaller value means that the value of the corresponding point of interest is biased toward negative, and a larger value means that the value of the point of interest is biased toward positive. The detection process of a single sample is divided into 3 stages. The first stage introduces the purpose and meaning of this visual expression of emotion to the test subject; the second stage, the test subject answers the first question, that is, the current emotional state. Afterward, the test subjects experience the visual expression system for emotions; in the third stage, the test subjects give the other four points of attention corresponding to the scores based on the second stage experience.

**TABLE 3:** Concerns and scores.

Number	Focus point	Score				
		1	2	3	4	5
1	Subject’s emotional state					
2	Recognition of the system					
3	Experience of the system					
4	Communication of system emotions					
5	Infection of systemic emotions					

## Experimental Results and Analysis

### Experimental Results

**FIGURE 7:**
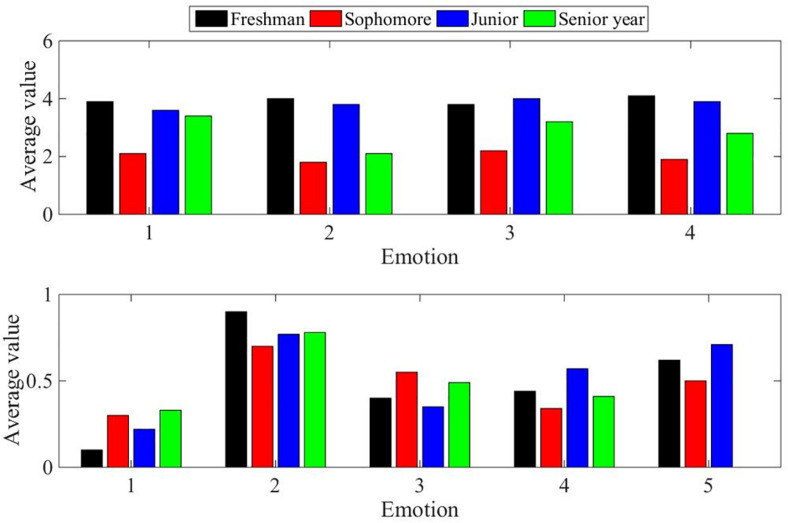
Mean and variance of the overall statistics.

After the completion of the test, a total of 100 valid questionnaires were collected. After the data was entered, the value of each focus point was counted, and the average value was calculated. At the same time, in order to verify the validity of the data, we also calculated the variance of all the data, and plotted the final statistics as shown in Figure 7.

### Experimental Analysis

**FIGURE 8:**
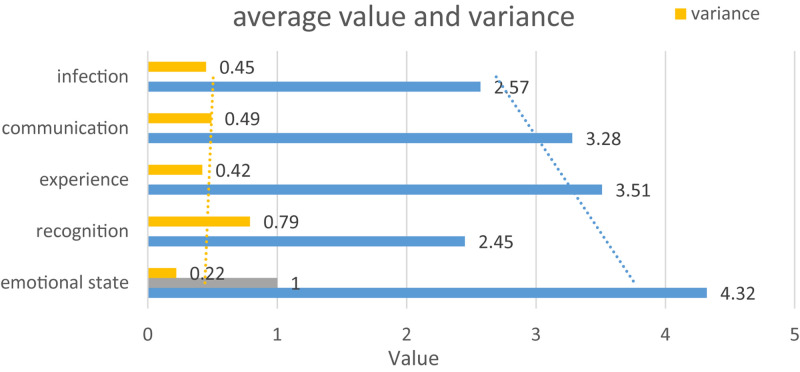
Mean and variance of overall statistics.

First, the statistical test is performed on whether there is a statistical difference in the answer results of the grades to the questionnaire, and the result is *p* > 0.05, which shows that there is no statistical significance, so the subsequent analysis is no longer for each grade. The overall data result is shown in Figure 8:

From the data obtained from the detection, we can see that the overall emotional state of the tested object before the detection is good, and from the variance data, it can be seen that there is not much difference in the overall distribution. In such an emotional state, it can be said that the detection data from the subject has a certain degree of authenticity. After the testee is tested, first analyze the data in the average statistics chart. It can be seen that in general, the testee does not accept this form of 3D dynamic website. The cause of the problem may be due to 3D testing. Although dynamic websites have emotional mechanisms, they have not yet been applied. Therefore, subjects cannot intuitively feel this form of emotional visualization. We also found that the variance value of the recognition statistics is obviously too large, which proves that there is a gap in the understanding of this concern among different test subjects. I believe that as long as this method of visual expression of emotions can be applied to specific counterattacks, more users can understand. Third, we analyze data related to user experience. In terms of the average value, it is found that the detected object is also in a positive evaluation overall, but its value does not reach a very high level. The reason is that as we analyzed before, it may be because of what we present to the detected object. The website lacks a clear support environment, which leads to a not very high experience. At the same time, it can also be seen that the variance value of the experience statistical data is generally high, indicating that there is a certain gap in the user experience of each measured object. We then analyze the concerns of transitivity and infectiousness. It can be seen from the average statistical graph that the values of these two focus points are generally positive. In other words, this kind of emotional three-dimensional dynamic website space has a certain ability to convey emotions, and the measured objects generally accept this attitude. At the same time, we also found that the variance of these two concerns is also in a high state, which means that different users have different understandings on this. This is actually in line with the characteristics of human beings, because the differences in feelings and understanding of emotions will always be different for each person. Therefore, we believe that this method of visual expression of emotion has the ability to convey or suggest emotion. Finally, we can summarize this test. Generally speaking, the data we collected is effective, and the emotion visualization methods discussed in this article can indeed convey or imply certain emotions, but we also found that there is a phenomenon that this recognition is not high enough, and the reason is analyzed: lack A practical application environment makes this emotional visualization lack of practical significance. At the same time, it can also be seen that different tested subjects have different feelings and evaluations of this kind of emotion visualization. As long as this difference is within a reasonable range, this kind of emotion visualization also has practical value.

## Conclusion

Human emotions have discrete and continuous characteristics. The construction of a comprehensive emotional synthesis model based on these two characteristics can describe human emotions more comprehensively. The qualitative and quantitative research methods of emotion in the comprehensive emotion synthesis model can model emotions more accurately to a certain extent. The visual expression based on the comprehensive emotional synthesis model makes the three-dimensional website present different forms, and the emotional interaction process between the website space and people is formed. Through the use of statistical analysis methods to detect the constructed emotional 3D dynamic website, the test results show that this emotion-based synthetic model can convey emotions to a certain extent, enhance the user experience of the dynamic 3D painting system, and enhance the superior experience of human-computer interaction.

## Data Availability Statement

The original contributions presented in the study are included in the article/supplementary material, further inquiries can be directed to the corresponding author/s.

## Author Contributions

SC: editing data curation and writing–original draft preparation.

## Conflict of Interest

The author declares that the research was conducted in the absence of any commercial or financial relationships that could be construed as a potential conflict of interest.

## Publisher’s Note

All claims expressed in this article are solely those of the authors and do not necessarily represent those of their affiliated organizations, or those of the publisher, the editors and the reviewers. Any product that may be evaluated in this article, or claim that may be made by its manufacturer, is not guaranteed or endorsed by the publisher.

## References

[B1] Andrew M. Larceneux F. (2019). The role of emotion in a housing purchase: an empirical analysis of the anatomy of satisfaction from off-plan apartment purchases in France. *Environ. Plan.* 51 1370–1388. 10.1177/0308518x18817539

[B2] Bringmann L. F. Pe M. L. Vissers N. Ceulemans E. Borsboom D. Vanpaemel W. (2016). Assessing temporal emotion dynamics using networks. *Assessment* 23 425–435. 10.1177/1073191116645909 27141038

[B3] Eyben F. Scherer K. R. Schuller B. W. Sundberg J. Andre E. Busso C. (2017). The Geneva Minimalistic Acoustic Parameter Set (GeMAPS) for Voice Research and Affective Computing. *IEEE Trans. Affect. Comput.* 7 190–202. 10.1109/taffc.2015.2457417

[B4] Fortin P. E. Cooperstock J. R. (2017). Laughter and tickles: toward novel approaches for emotion and behavior elicitation. *IEEE Trans. Affect. Comput.* 8 508–521. 10.1109/taffc.2017.2757491

[B5] Hu X. Chen J. Wang F. Zhang D. (2019). Ten challenges for EEG-based affective computing. *Brain Sci. Adv.* 5 1–20. 10.1177/2096595819896200

[B6] Huang A. (2018). A risk detection system of e-commerce: researches based on soft information extracted by affective computing web texts. *Electron. Commer. Res.* 18 143–157. 10.1007/s10660-017-9262-y

[B7] Huxtable-Thomas L. A. Hannon P. D. Thomas S. W. (2016). An investigation into the role of emotion in leadership development for entrepreneurs: a four interface model. *Int. J. Entrep. Behav. Res.* 22 510–530. 10.1108/ijebr-12-2014-0227

[B8] Kim D. Jeon J. Cheong E. Kim D. J. Ryu H. Seo H. (2016). Neuroanatomical Visualization of the Impaired Striatal Connectivity in Huntington’s Disease Mouse Model. *Mol. Neurobiol.* 53 2276–2286. 10.1007/s12035-015-9214-2 25976370

[B9] Kostelnick C. (2016). The re-emergence of emotional appeals in interactive data visualization. *Tech. Commun.* 63 116–135.

[B10] Liu H. Min X. Wang J. Rao T. Burnett I. (2016). Improving Visual Saliency Computing With Emotion Intensity. *IEEE Trans. Neural Netw. Learn. Syst.* 27 1201–1213. 10.1109/tnnls.2016.2553579 27214350

[B11] Liu Y. Fu G. (2021). Emotion recognition by deeply learned multi-channel textual and EEG features. *Future Gener. Comput. Syst.* 119 1–6. 10.1016/j.future.2021.01.010

[B12] Qiu J. L. Zhao W. Y. (2018). Data Encoding Visualization Based Cognitive Emotion Recognition with AC-GAN Applied for Denoising// IEEE International Conference on Cognitive Informatics & Cognitive Computing. *IEEE Comput. Soc.* 7 222–227.

[B13] Radojevic N. (2016). Perspective, Cartography and Dynamic Notions: From the Plane Bozzetto to Solid Perspective in Two Examples in Tuscany. *Nexus Netw J.* 18 669–695. 10.1007/s00004-016-0300-1

[B14] Ren F. Matsumoto K. (2017). Semi-automatic creation of youth slang corpus and its application to affective computing. *IEEE Trans. Affect. Comput.* 7 176–189. 10.1109/taffc.2015.2457915

[B15] Ridderinkhof K. R. (2017). Emotion in Action: A Predictive Processing Perspective and Theoretical Synthesis. *Emot. Rev.* 9 319–325. 10.1177/1754073916661765 29098017 PMC5652650

[B16] Tang Y. Elhoseny M. (2019). Computer network security evaluation simulation model based on neural network. *J. Intell. Fuzzy Syst.* 37 1–8. 10.3233/JIFS-179121

[B17] Topal K. Ozsoyoglu G. (2017). Emotional classification and visualization of movies based on their IMDb reviews. *Inf. Disc. Deliv.* 45 149–158. 10.1108/idd-05-2017-0045

[B18] Ueoka R. AlMutawa A. (2018). “Emotion Hacking VR: Amplifying Scary VR Experience by Accelerating Actual Heart Rate,” in *Human Interface and the Management of Information. Interaction, Visualization, and Analytics*, Vol. Vol. 10904 eds Yamamoto S. Mori H. (Berlin: Springer Verlag), 436–445. 10.1007/978-3-319-92043-6_37

[B19] Volante M. Babu S. V. Chaturvedi H. Newsome N. Ebrahimi E. Roy T. (2016). Effects of virtual human appearance fidelity on emotion contagion in affective inter-personal simulations. *IEEE Trans. Vis. Comput. Graph.* 22 1326–1335. 10.1109/tvcg.2016.2518158 26780808

[B20] Wang Q. Lu P. (2019). Research on Application of Artificial Intelligence in Computer Network Technology. *Intern. J. Pattern Recognit. Artif. Intell.* 33:1959015. 10.1142/s0218001419590158

[B21] Wang Y. Segal A. Klatzky R. Keefe D. F. Isenberg P. Hurtienne J. (2019). An emotional response to the value of visualization. *IEEE Eng. Med. Biol. Mag.* 39 8–17. 10.1109/mcg.2019.2923483 31442961

[B22] Yu J. Li L. Zou J. (2017). Realistic emotion visualization by combining facial animation and hairstyle synthesis. *Multimed. Tools Appl.* 76 14905–14919. 10.1007/s11042-016-4239-8

[B23] Zhang X. Liu J. Shen J. Li K. Hou B. Hu J. (2020). Emotion recognition from multimodal physiological signals using a regularized deep fusion of kernel machine. *IEEE Trans. Cybern.* 1–14. 10.1109/tcyb.2020.2987575 32413939

[B24] Zhang Y. Chen J. Liu B. Yang Y. Li H. Zheng X. (2020). COVID-19 public opinion and emotion monitoring system based on time series thermal new word mining. *Comput. Mater. Continua.* 64 1415–1434. 10.32604/cmc.2020.011316

[B25] Zhou P. Li K. Wei W. Wang Z. Zhou M. (2020). Fast generation method of 3D scene in Chinese landscape painting. *Multimed. Tools Appl.* 79 16441–16457. 10.1007/s11042-019-7476-9

[B26] Zhu X. Xue L. (2020). Building a controllable expressive speech synthesis system with multiple emotion strengths. *Cogn. Syst. Res.* 59 151–159. 10.1016/j.cogsys.2019.09.009

